# Right-sided Bochdalek hernia in an elderly adult: a case report with a review of surgical management

**DOI:** 10.1186/s40792-017-0385-0

**Published:** 2017-10-13

**Authors:** Kazuki Moro, Mikako Kawahara, Yusuke Muneoka, Yu Sato, Chie Kitami, Shigeto Makino, Atsushi Nishimura, Yasuyuki Kawachi, Emmanuel Gabriel, Keiya Nikkuni

**Affiliations:** 1 Department of Surgery, Institute of Gastroenterology, Nagaoka Chuo General Hospital, Nagaoka, Niigata 940-8653 Japan; 2 Section of General Surgery, Department of Surgery, Mayo Clinic, Jacksonville, FL 32224 USA

**Keywords:** Bochdalek hernia, Strangulation, Adult, Right-sided

## Abstract

**Background:**

Bochdalek hernias are one of the most common types of diaphragmatic hernia, with most cases diagnosed during the neonatal period. In contrast, diagnosis of a Bochdalek hernia in an adult is rare and is typically observed on the left side of the diaphragm. Even more rare is the diagnosis of a right-sided Bochdalek hernia in an adult, where there is concurrent visceral malformation in most cases.

**Case presentation:**

We describe a case of an 89-year-old female who presented with abdominal pain. An abdominal computed tomography (CT) scan showed decreased intravenous contrast uptake and thickening of the wall of herniated small intestine through the right side of the diaphragm, which led to the diagnosis of a strangulated diaphragmatic hernia. The patient underwent emergent laparotomy and required a partial resection of the necrotic ileum and a hernia repair with direct closure. Interestingly, in this case, organ malformation was not observed. The patient was discharged approximately 2 weeks after surgery without complication.

**Conclusions:**

Adult right-sided Bochdalek hernia with strangulation in the absence of hepatic atrophy is a rare entity. Considering the severity of this condition, accurate diagnosis and proper treatment are needed. A tailored operative approach is required on an individual case basis.

## Background

Bochdalek hernia is a diaphragmatic hernia usually diagnosed during the neonatal period. It typically occurs in the left hemi-diaphragm and presents with severe respiratory and circulatory compromise. Thus, the mortality rate is high. Adult Bochdalek hernia is rare, and most are also found on the left side of the diaphragm because the right pleuroperitoneal canal closes earlier and the liver buttresses the right diaphragm, minimizing the opportunity for herniation into the right thoracic cavity [1–3]. However, as a corollary to this rationale, organ malformation consisting primarily of hepatic atrophy can increase the risk of a right-sided diaphragmatic hernia. Few of these rare cases have been reported.

An adult Bochdalek hernia is usually precipitated by a state of increased intra-abdominal pressure, such as that induced by pregnancy or from prolonged operations under pneumoperitoneum [4]. Thus, as the number of laparoscopic-assisted operations increases, it is important to consider a Bochdalek hernia as a potential intraoperative or postoperative complication. Iatrogenic weakness of the diaphragm that occurs as a result of abdominal surgery, such as hepatectomy, esophagogastrectomy, or transthoracic hiatal hernia repairs, may also cause an adult Bochdalek hernia [5, 6]. In light of the possibility of a diaphragmatic hernia being acquired, it is difficult to determine the exact cause of an adult Bochdalek hernia. Regarding the pathophysiological process that produces a congenital defect of the diaphragm, Chui et al. reported that such a defect may be exacerbated by elevations in intra-abdominal pressure, which consequently results in a hernia [7]. Thus, this diaphragmatic defect may confer a predisposition to the development of a hernia in the context of increased intra-abdominal pressure. Here, we present an adult case of a right-sided Bochdalek hernia associated with strangulation ileus, in the absence of organ malformation, followed by a brief review of its diagnosis and management.

## Case presentation

An 89-year-old female with a previous surgical history of a uterine myoma resection, right femur fracture, and right femoral hernia repair presented with abdominal pain and dyspnea at an outside institution. She had a history of two vaginal deliveries and no history of upper abdominal surgery. After an open operation for a right femur fracture when she was 87 years old, her physical activity decreased. This led to decreased appetite and weight loss. Her body mass index (BMI) was 14.0 on admission.

She required an emergent admission with respiratory monitoring and supplemental oxygen for low saturation. She was observed for 4 days. She had an ileus during this time, which was diagnosed by abdominal X-ray, and an ileus tube was placed. Her treating physicians requested a transfer to our institution. A physical examination on admission showed a mildly protuberant abdomen. Laboratory tests, including leukocyte count and electrolyte, were normal. The serum C-reactive protein (CRP) was mildly elevated to 0.46 mg/dl (normal range 0.01–0.30). A lactic acid level and an arterial blood gas were both normal.

**Fig. 1:**
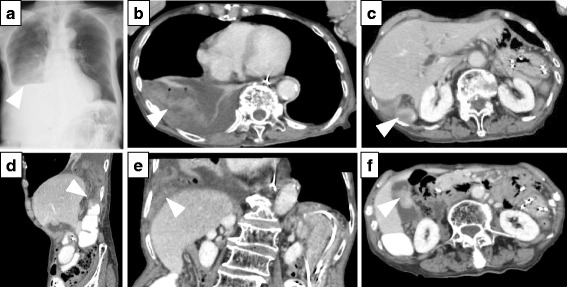
Radiographic images of the adult right-sided Bochdalek hernia prior to surgery. **a** Chest X-ray showed intestinal gas over the liver and an elevated right hemi-diaphragm (white arrow head). **b** A computed tomography (CT) scan of the abdomen and pelvis demonstrated reduced contrast uptake and thickening of the herniated small intestinal wall. The herniated small intestine was present in the thorax (white arrow head). **c** CT scan of the abdomen and pelvis demonstrated herniation of the small intestine into the right thoracic cavity from the posterior surface overlying the right hepatic lobe (white arrow head). It also demonstrated reduced contrast uptake and thickening of the herniated small intestinal wall. The liver was not atrophic. **d** A sagittal sequence of CT scans confirmed herniation of the small intestine into the right thoracic cavity from the posterior surface overlying the right hepatic lobe (white arrow head). **e** A coronal sequence of CT scans also demonstrated herniation of the small intestine into the right thoracic cavity from the posterior surface overlying the right hepatic lobe (white arrow head). **f** CT scan of the abdomen and pelvis also demonstrated a torose lesion in the gallbladder (white arrow head)

Chest X-ray demonstrated intestinal gas over the liver and an elevated right diaphragm (Fig. 1a). A computed tomography (CT) scan of the abdomen and pelvis demonstrated reduced contrast uptake and thickening of the herniated small intestinal wall. The herniated small intestine was present in the thorax, as demonstrated by the lower cuts of the abdominal CT scan (Fig. 1b). The findings of reduced intravenous (IV) contrast uptake and thickening of the bowel wall led to the diagnosis of a strangulated hernia through the right hemi-diaphragm. Specifically, the CT scan demonstrated herniation of the small intestine into the right thoracic cavity through the posterior surface overlying the right hepatic lobe (white arrow head). The liver was not atrophic (Fig. 1c–e). The patient, therefore, underwent emergent surgery.

**Fig. 2:**
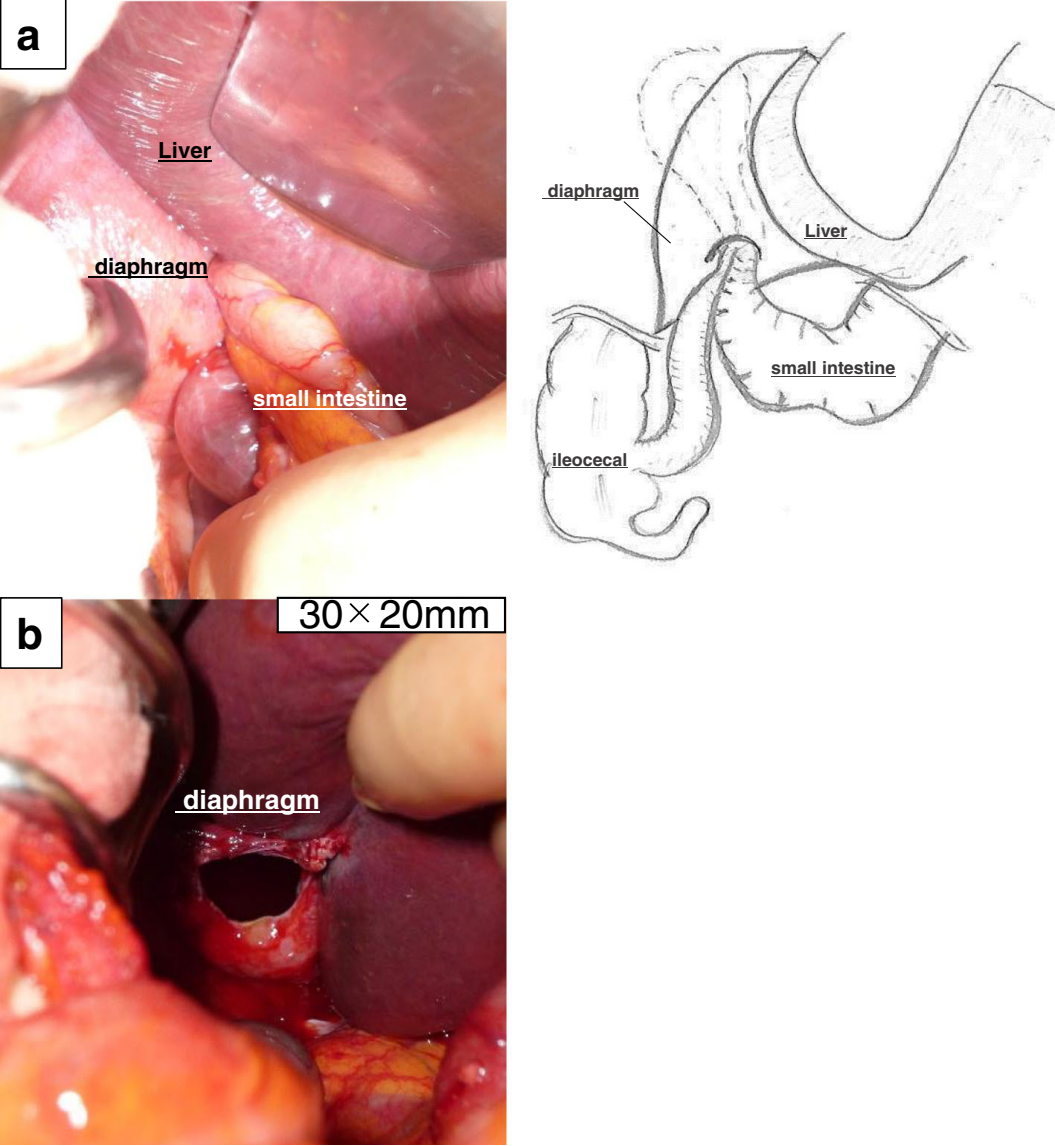
Surgical approach to repair of the right-sided Bochdalek hernia. **a** Presence of an oval hernia orifice with a diameter of 3 cm in the right posterior surface of the diaphragm. **b** The small intestine was reduced back into the abdominal cavity from the right thoracic cavity, demonstrating the diaphragmatic hernia defect

Considering the likely probability of necrotic small bowel, we selected an abdominal approach to the hernia. Surgical findings at laparotomy confirmed the presence of an oval hernia defect with a diameter of 3 cm in the right posterior surface of the diaphragm (Fig. 2a). The ileum was necrotic over an area of the 30 cm in length and surrounded by a reactive pleural effusion. However, the bowel was not grossly perforated. The hernia contents were reduced into the abdominal cavity from the right thoracic cavity (Fig. 2b). The diaphragmatic defect was closed primarily with triclosan-coated absorbable surgical suture (0 Vicryl), with adequate exposure following partial mobilization of the right liver. The necrotic ileum was resected, and we performed a side-to-side, functional end-to-end stapled anastomosis. We performed a lavage of the right chest cavity. We selected not to place a drain in the right chest cavity, because the field was not grossly contaminated.

In addition, the CT scan of the abdomen and pelvis also demonstrated papillary lesion in the gallbladder suspicious for malignancy, and so, a cholecystectomy was performed (Fig. 1f). We decided not to perform a lymph node dissection. Because this was an emergent case in an elderly patient, we elected to make the operation time as short as possible. The surgery lasted 124 min, and blood loss was 22 ml.

**Fig. 3:**
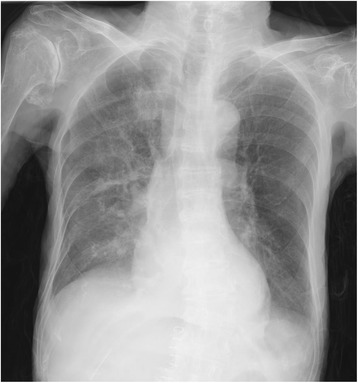
Radiographic image of the adult Bochdalek hernia after operation. Chest X-ray showed no recurrence

A post-operative chest X-ray was performed on day 3, which showed no recurrence (Fig. 3). The patient recovered without any post-operative complications and was discharged on the 13th day after surgery. The gallbladder pathology was papillary adenocarcinoma stage T1a. Thus, additional treatment was considered unnecessary.

### Discussion

Adult Bochdalek hernia commonly presents with gastrointestinal symptoms such as abdominal pain, abdominal distension, and vomiting due to obstruction of the prolapsed gastrointestinal tract [8]. Although the adult lungs are fully developed, the symptom of impaired respiration because of adult Bochdalek hernia can still occur. The prolapsed organ may not only involve the gastrointestinal tract but may also include the liver in right-sided adult Bochdalek hernia. As in our case, strangulation of the herniated intestine can occur. If the prolapsed small bowel undergoes necrosis or perforation, the condition may be fatal. Although adult Bochdalek hernia has the potential to be a very morbid condition, the diagnosis can be made with careful radiographic examination. As in our case, a chest X-ray showing intestinal gas over the liver and the elevated diaphragm was highly suspicious for a diaphragmatic hernia.

Adult right-sided Bochdalek hernia is a rare disease, often associated with organ malformation such as an atrophic liver. Mullins et al. retrospectively reviewed 13,138 abdominal CT scans and diagnosed this entity in 22 patients, which represented an incidence of 0.17% [9]. To the best of our knowledge, there have been 14 reported cases of right-sided adult Bochdalek hernia in Japan, which are summarized in Table 1. The average age at diagnosis is 61.1 years old. It has been reported that several organ malformations, such as universal mesentery, malrotation, volvulus of the stomach, and liver atrophy, are accompanied with right-sided Bochdalek hernia. In 11 of 14 cases, malformation of organs was observed. Interestingly, in the present case, there was no associated organ malformation.

**Table 1:** Summary of the reported cases of adult right sided Bochdalek hernia in Japan

Case	Author	Year	Age	Gender	Chief complaint	Surgical approach	Malformation	Hernia orifice	Closure
1	Ochi	1984	43	Male	Abdominal pain	Laparotomy	Volvulus of the stomach	“Egg size”	Suture
2	Zaima	1984	47	Female	Chest pain	Laparotomy	Mesenterium commune	4 × 6 cm	Suture
3	Itoda	1997	68	Female	Epigastric pain	Laparotomy	Right liver atrophy	8 × 5 cm	Suture
4	Zenda	2000	69	Male	Epigastric pain	Laparotomy	Left liver atrophy	10 × 15 cm	Suture
5	Kanazawa	2000	63	Female	Abdominal pain	Laparotomy	None	12 cm	Suture
6	Kiriyama	2002	41	Female	Cough, sputum	Laparotomy	Intestinal malrotation Right liver atrophy	10 × 7 cm	Mesh
7	Kato	2004	55	Female	Right hypochondriac pain	Laparotomy	Right kidney malposition	8 × 8 cm	Mesh
8	Masuda	2007	80	Female	Abdominal pain	Laparotomy	Incomplete fixation of the duodenum	Unknown	Suture
9	Matsushita	2009	21	Female	No complaints	Thoraco-laparotomy	None	3 cm	Suture
10	Murakami	2010	85	Male	Abdominal pain	Laparotomy	Right liver atrophy	3 cm	Suture
11	Nishiwaki	2011	59	Male	Bloody stool	Thoracotomy	Right liver atrophy	10 × 8 cm	Mesh
12	Mizoguchi	2013	71	Male	Dyspnea	Laparotomy	Mesenterium commune	4 × 3.5 cm	Mesh
13	Watanabe	2015	65	Female	Abdominal pain	Laparotomy	Right liver atrophy	5 cm	Suture
14	Moro	2017	89	Female	Abdominal pain	Laparotomy	None	3 × 2 cm	Suture

In this current case, the patient had some elements in her history that may have caused the elevation of intra-abdominal pressure. She had a history of two vaginal deliveries and ileus with right femoral hernia strangulation. Given that the patient’s BMI was low, there may also have been inherent thinning of the diaphragm. Although the cause of our patient’s hernia is unclear, given the patient’s age and laxity of tissues in conjunction with the history of a previous hernia, the patient may have been considered at an increased risk of this condition.

In treating adult Bochdalek hernia, it is important to perform surgical closure of the hernia orifice and reduction of the prolapsed organs. There is no established consensus for choosing an approach, although the approach should be selected on a case-by-case basis. Transabdominal, transthoracic, and combined thoracoabdominal routes are different approaches [10, 11]. There are advantages and disadvantages for each approach. The laparotomy is beneficial with respect to facilitating intra-abdominal manipulation of reduced abdominal organs (such as the ileum in our case), observation of perfusion defects in prolapsed organs, and management of injury to the reduced organs. Conversely, an abdominal approach limits exposure to the chest cavity and can result in a persistent pneumothorax or pleural effusion [10]. The thoracotomy is beneficial with respect to repair of the hernia orifice, particularly in the case of a right-sided defect due to a direct, usually unobstructed visualization of the diaphragm [12]. In contrast, in an abdominal approach to a right-sided diaphragmatic hernia, the liver may require mobilization if no atrophy is present. A thoracoabdominal approach provides maximum exposure in both the thoracic and abdominal cavities, though this approach can be quite morbid. In the present case, given the necessity of partial resection of the necrotic segment of ileum, we performed the transabdominal approach and confirmed whether the organ malformation existed. Although there are some reports of thoracoscopic and laparoscopic surgery for treatment of Bochdalek hernia [13–16], in right-sided Bochdalek hernia, this approach may prove to be difficult because of deep and high operative field over the liver, the emergent nature of the operation, and a narrow laparoscopic working space.

The diaphragmatic defect itself is typically repaired by simple suture closure, implantation of a prosthetic mesh repair, or use of a muscle flap [17]. The approach and selection of the suture/mesh are performed on a case-by-case basis. An absorbable suture has the advantage of minimizing the infection risk. On the other hand, the non-absorbable suture has the advantage of preventing recurrence [18]. When the hernia orifice is large or the tissues are very friable and not amenable to suture closure, it is important to select a mesh repair or muscle flap. Sato et al. reported that 5 cm is the upper limit length for suture closure, and there have been cases of recurrence with hernia orifices of this size [19]. In this case, given that the defect was under 5 cm, the advanced age of the patient, and the possibility of infection with increased morbidity for this patient, we performed simple suture closure with the triclosan-coated absorbable suture.

## Conclusions

Considering that almost all cases of adult right-sided Bochdalek hernia have organ malformation (liver atrophy), our presented case is quite atypical. Diagnosis can be firmly established with diagnostic radiology and prompt surgical intervention is required to a favorable outcome. The surgical approach can be considered on an individualized basis, though laparotomy offers a more optimal exposure for resection of non-viable bowel.

## Abbreviations

BMI: Body mass index
CRP: C-reactive protein
CT: Computed tomography
IV: Intravenous
